# Vascularised Composite Allotransplantation: Emerging Applications in Reconstructive Surgery and Solid Organ Transplantation

**DOI:** 10.3390/medicina62020245

**Published:** 2026-01-23

**Authors:** Cian M. Hehir, Michael O’Connor, Iulia Marinescu, Fungai Dengu, Henk P. Giele, Roisin T. Dolan

**Affiliations:** 1 Tissue Engineering Research Group (TERG), Royal College of Surgeons in Ireland, 123 St. Stephen’s Green, D02 YN77 Dublin, Ireland; 2 Department of Surgery, Royal College of Surgeons, 123 St. Stephen’s Green, D02 YN77 Dublin, Ireland; 3 Department of Plastic & Reconstructive Surgery, St. Vincent’s University Hospital, Elm Park, D04 T6F4 Dublin, Ireland; 4 UCD School of Medicine & Medical Sciences, University College Dublin, D04 V1W8 Dublin, Ireland; 5 Department of Hepatobiliary & Transplant Surgery, St. Vincent’s University Hospital, Elm Park, D04 T6F4 Dublin, Ireland; 6 Department of Plastic & Reconstructive Surgery, Oxford University Hospitals NHS Foundation Trust, University of Oxford, Oxford OX3 9DU, UK; 7 Oxford Research in Plastic Surgery and Hand Surgery Innovation Collaboration (ORPHIC), Nuffield Department of Surgical Sciences, University of Oxford, Oxford OX3 9DU, UK

**Keywords:** vascularised composite allotransplantation (VCA), allograft, reconstructive microsurgery, sentinel skin flap (SSF)

## Abstract

Vascularised composite allotransplantation (VCA) has an evolving role in the reconstruction of complex functional and aesthetic deficits non-amenable to autologous or implant-based reconstructive modalities. International applications of VCA span upper extremity, face, abdominal wall, uterus, and penile transplantation, with more than 300 procedures performed worldwide. Among these, abdominal wall transplantation has uniquely contributed to the development of the sentinel skin flap (SSF) concept, in which solid organ transplant patients undergo simultaneous transplantation of a solid organ and a donor-derived vascularised skin flap, with the skin component of the SSF being trialled internationally as a means of monitoring for rejection within the solid organ allograft. Despite growing clinical success, VCA continues to face substantial barriers to wider adoption. Acute rejection remains highly prevalent, affecting up to 89% of recipients, with significant morbidity linked to intensive systemic immunosuppression. Challenges are further amplified by the unique immunological heterogeneity of composite grafts, ethical concerns surrounding identity-linked tissues, and the lack of standardised outcomes reporting across VCA subtypes. Advances in machine perfusion technologies and emerging cellular and biomaterial-based immunomodulation strategies show promise in reducing immunosuppression burden and improving graft longevity. This review outlines the current state of VCA, including clinical applications, outcomes, and mechanistic insights from pre-clinical studies, while highlighting key ethical considerations and evolving regulatory frameworks. Future progress will depend on standardised reporting systems, improved donor–recipient matching, better understanding of ischemia–reperfusion injury, and the development of next-generation immunosuppressive/immuno-modulatory therapies. Collectively, these innovations position VCA as a rapidly advancing field with significant potential to redefine reconstructive and transplant surgery.

## 1. Introduction

Vascularised Composite Allotransplantation (VCA) involves the transplantation of composite tissue allografts, as a functional unit, between an allo-incompatible donor and the recipient, with grafts typically involving a skin or mucosal component [1]. This specialised procedure has enabled significant advancements in reconstructive surgery, providing life-altering functional and aesthetic restoration to patients who have suffered significant tissue loss. VCA encompasses a broad range of composite tissue grafts which, when transplanted as entire subunits, offer unique functionally restorative applications, ranging from skin-containing hand, face, and abdominal wall transplants to uterine and penile allotransplantation [2,3,4,5]. The field of VCA has emerged as a solution to the limitations of conventional reconstructive techniques, such as autologous tissue transfer and prosthetics, which are often deficient in achieving full restoration of both the functional and aesthetic aspects of complex tissue defects [6,7].

Abdominal wall transplantation (AWT) VCA, aimed at providing primary abdominal closure following intestinal and/or multi-visceral organ transplantation, was first described in the literature in the early 2000s [8]. In intestinal/multi-visceral abdominal organ transplantation, closure is often complicated by tissue deficiency owed to prior laparotomy, ostomy construction, enterocutaneous fistulae, or abdominal wall fibrosis [8,9]. Although the initial aim of AWT was to achieve abdominal closure, a secondary function of the abdominal wall VCA later emerged. Gerlach et al. first reported the potential for the skin component of AW VCA to act as a ‘sentinel’ marker of immunological activity within the visceral allograft, termed thereafter as a Sentinel Skin Flap (SSF) transplant [10]. The high immunogenicity of skin, its external visibility, and its capacity to rapidly demonstrate skin changes such as erythema and soft-tissue oedema during rejection episodes, presents an attractive possible means of monitoring for the immunologically mediated rejection of visceral grafts [11]. Timely diagnosis of allograft rejection is essential to limit rejection-mediated morbidity and graft destruction, with delayed diagnosis associated with significantly inferior graft outcomes [12,13,14]. As such, international efforts have focused on the identification of serum biomarkers capable of accurately diagnosing allo-rejection prior to allograft injury. A number of candidate biomarkers have demonstrated clinical promise, such as ‘liquid biopsy’, aimed at identifying the presence (or absence) of donor-derived cell-free DNA in the recipient serum, a known marker of allograft destruction [15]. Liquid biopsy demonstrates a high negative predictive value (90–97%), rendering it clinically useful in the exclusion of allograft rejection [16]. However, the role of liquid biopsy remains limited in the diagnosis of allo-rejection, owing to its relatively low positive predictive value (33–56%) [16]. Further biomarkers of interest include CXCL-10, KIM-1, and NGAL. Despite the development of such biomarkers, transplant biopsy remains the gold standard in the diagnosis of rejection [17,18]. Although the development of more sensitive and specific rejection biomarkers represents significant progress in transplantation, the utility of serum markers relies fundamentally on patients physically presenting for assessment. SSF may offer a plausible solution to graft surveillance in the community, presenting potential avenues for both the remote physician monitoring and patient lead monitoring of sentinel grafts. Sentinel skin flap transplantation has since evolved to include broader applications than AWT, with current randomised control trials utilising smaller SSFs, such as the radial forearm free flap utilised in the SENTINEL trial, which aims to evaluate SSF in lung transplantation [19].

Significant progress has been made in VCA, spanning both pre-clinical and clinical domains alike [20]. The first successful hand transplant in 1998 and the first facial transplant in 2005 marked critical milestones in the field, paving the way for subsequent advances in microsurgical techniques, immunosuppressive protocols, and patient selection criteria [21,22]. However, a number of factors continue to impede the broader clinical application of VCA. The requirement for lifelong immunosuppression and the associated increased infective, neoplastic, and lymphoproliferative morbidity risk represents a significant barrier to VCA [23]. Although systemic immunosuppression is a fundamental requirement in solid organ transplantation (SOT), the risk–benefit to patients warrants more careful consideration in VCA, where the primary aim is that of functional and anatomical restoration as opposed to life-saving intervention for patients with end-stage organ disease, as in SOT. Despite systemic immunosuppression at similar or higher concentrations to that in SOT, the rate of acute and chronic allograft rejection remains disappointingly high, further emphasising the necessity for innovative immunomodulatory strategies within the field [24,25]. The psychological impact of VCA, both in terms of patient identity and donor consent, further complicates ethical considerations surrounding the procedure [26,27]. Although there are common barriers to donation, which traverse both SOT and VCA, the externally visibility of VCA grafts and the associated fear of ‘identity loss’ remain commonly cited barriers among those who oppose VCA donation [28].

In recent years, VCA research has focused on improving allograft survival rates, the minimisation of ischaemic reperfusion-mediated allograft injury, and the development of novel immune tolerance strategies [29,30,31]. Efforts to establish standardised regulatory frameworks and to expand accessibility to VCA remain critical for integrating these procedures into routine clinical practice. This review aims to outline the current applications, clinical outcomes, and challenges facing VCA. We explore the latest advancements, ethical considerations, and future directions of the field.

## 2. Current Applications

As of 2024, a total of 300 vascularised composite allotransplantations (VCAs) have been performed worldwide, including 148 upper extremity transplants, 80 uterine transplants, 48 face transplants, 46 abdominal wall transplants, five penis transplants, and two lower extremity transplants [32]. The initial OPTN/SRTR report revealed that, between 2014 and 2021, 31 uterus and 49 non-uterus VCA transplants were performed in the United States. The most transplanted VCA was the upper limb (48%), followed by the face (35%). Trauma (45%), infection (26%), and burn/explosion injuries (6%) were the leading causes necessitating VCA. The majority of recipients were male (74%), with the most common age group being 18–34 years (39%) [33]. The growing number of live births rendered possible through uterine transplantation speaks volumes for the potential of vascularised composite allotransplantation in achieving meaningful functional restoration [34]. Upper limb transplantation has witnessed similar patient-reported success with upper limb VCA recipients reporting a mean improvement in Disability of the Arm, Shoulder, and Hand (DASH) Score of 32.0 (*p* < 0.001) [35]. This demonstrates the significant capacity for upper extremity VCA to deliver functional restoration to patients. Although the functional outcomes of these grafts shed a hopeful light on the progress and potential of VCA, standardised reporting of outcomes is still lacking within this evolving clinical entity, rendering critical appraisal difficult on a global scale.

## 3. Clinical Outcomes

In 2020, the American Organ Procurement and Transplantation Network (OPTN) and the Scientific Registry of Transplant Recipients (SRTR) began including VCA patient data in their annual transplantation report. Although outcomes were limited to graft survival versus failure alone, this is an essential first step toward standardisation of reporting in VCA. Expert stakeholders in uterine VCA have emerged as the first to propose standardised reporting variables and define transplantation success within their VCA subdivision [36]. Preferred reporting of outcomes would certainly vary depending on the VCA subtype in question. This is especially relevant with respect to functional outcomes. Establishing a common metric for VCA functional success has proven difficult. Primarily, this difficulty is a result of the disparate functions of various VCA subtypes, rendering comparison with a common metric fundamentally invalid. Functional outcome measures would benefit from specificity to the VCA in question, such as DASH in upper limb VCA, which has been specifically validated for upper extremity functional assessment, with the included tasks being directly associated with patient-reported quality of life [37].

Although the functional viability of transplanted allografts is of definite importance, the immunological tolerance or hypo-reactivity of transplanted VCAs is fundamental to a successful post-transplant clinical course. A systematic review analysing the clinical course of 115 pooled VCA recipients from 1998 to 2021 reported that 89% of recipients experienced at least one episode of acute rejection, whereas 11% developed chronic rejection. Unfortunately, this resulted in 19% (11% facial and 22% upper-extremity) of VCAs being explanted with a mean time to explantation of 26.9 months. Complications associated with systemic immunosuppression were reported, with infectious (58%), metabolic (hyperglycaemia 47%, hypertension 17%, and hyperlipidaemia 13%), and malignant (7%) morbidity being among the most commonly reported [38]. Immediate surgical complications were also reported, with anastomotic complications being the most prevalent (11% facial, 17% upper-extremity, and 15% total). Fistulation was a relatively common complication of facial transplantation (7%), but did not complicate upper limb allotransplantation. There was a low rate of haematoma (3%) and seroma (1%) across facial and upper limb transplantation [38]. Lower extremity transplantation remains experimental, with inferior functional outcomes and surgical complications compared to that of upper extremity VCA [39]. The post-operative functional gains have been demonstrated as inferior in lower extremity VCA, with two of the patients who underwent lower extremity VCA reported as deceased within 6-months post-operatively [39]. A further one patient suffered significant morbidity and required explantation due to an immunosuppression-related CNS lymphoma [39].

Patient reported outcome measures (PROMs) are of critical importance in defining success in VCA. Despite several authors highlighting the need for the development of VCA-specific PROMs, the utilised outcome measures remain heterogenous in the literature [40,41]. Given that the primary aims of VCA centre around functional restoration and quality of life enhancement, it is sensical that the ideal VCA PROMs would evaluate these areas. Some authors highlight that these outcome measures alone fail to capture key challenges inherent in the post-transplant experience in VCA, which include the physiological, psychological, and financial burden of lifelong immunosuppression [42]. Others highlight the onerous rehabilitation course following VCA as requiring further exploration [41].

## 4. Challenges Facing VCA

The core challenges facing VCA are of an immunological origin. The immunogenicity of transplanted tissue is known to vary depending on the tissue type [43]. As such, the heterogenous combination of tissues, which constitute VCAs, present a complex immunological picture. Enhanced understanding of allorecognition, alloreactivity, and molecular mechanisms of rejection in VCA is essential if allograft rejection rates are to be improved. This section details the critical immunological aspects of VCA.

### 4.1. Immunology in VCA Allorecognition and Alloresponse Mechanisms

**Figure 1:**
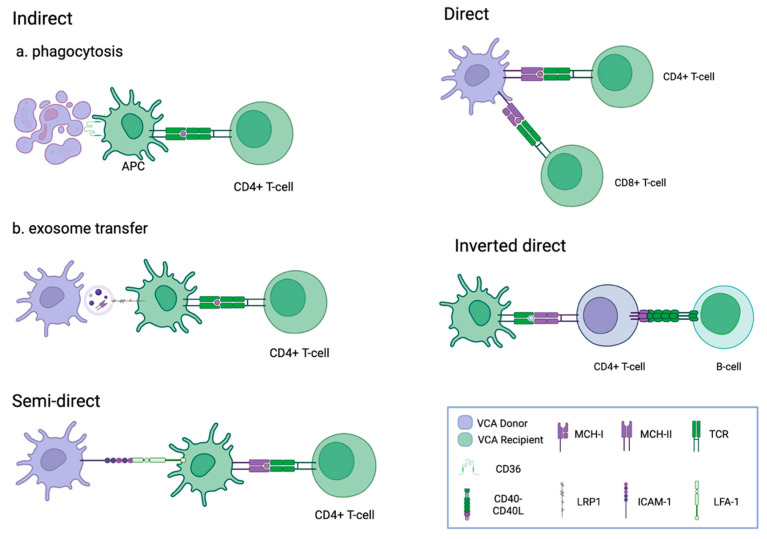
Modes of allorecognition in allotransplantation. In direct allorecognition, donor MHC I & II molecules on the surface of donor APCs are recognised as alloantigens by recipient T-lymphocytes. Inverted direct allorecognition, as the name would suggest, is inverse to the direct allorecognition pathway from a cellular perspective and involves the activation of passenger donor T-lymphocytes by MHC II molecules on the surface recipient APCs. Indirect allorecognition involves internalisation and the expression of donor alloantigens by recipient APCs through phagocytosis (**a**) or exome transfer (**b**) and are presented to recipient T-lymphocytes by recipient APCs. Semi-direct allorecognition involves the transfer of donor MHC II molecules to recipient APCs, which ultimately activate recipient T-lymphocytes. Created in BioRender. Marinescu, I. (2025) https://BioRender.com/q39a521, accessed on 18 January 2026.

The cellular heterogeneity of composite allografts presents an immunological challenge for VCA, especially in skin- and mucosa-containing allografts, which are known to be highly immunogenic [43,44,45]. Acute rejection (AR) remains the most common immunological complication in VCA and SOT alike. Although AR can be treated effectively with rescue immunosuppressive treatments, the number and severity of AR episodes are predictive for chronic allograft rejection and, ultimately, increased risk of graft loss [24,46]. AR is primarily T-lymphocyte-mediated and occurs when donor-specific alloantigen is presented by resident antigen presenting cells (APCs) to T-lymphocytes through direct, inverse-direct, indirect, and semi-direct allorecognition mechanisms [47] (Figure 1). Direct allorecognition involves the presentation of intact donor MHC–peptide complexes by donor APCs to recipient T-lymphocytes (Figure 1). Contrastingly, the indirect allorecognition pathway involves both APC and T-lymphocytes, which are of recipient origin. Donor proteins are processed into peptides by recipient APCs through phagocytosis or exosome transfer and are presented on recipient MHC-II molecules for allorecognition by recipient T-cells (Figure 1). Inverted direct and semi-direct pathways are mechanistically similar, both involving the presentation of intact donor MHC molecules by recipient APCs (Figure 1). Additionally, the semi-direct pathway processes and presents donor antigens on the intact donor MHC (Figure 1).

Following allorecognition, alloreactive T-lymphocytes undergo rapid division and differentiation into effector T-cells (T_EFF_), with resultant alloantigen-specific destruction of donor tissues. The density of donor APCs in VCA, particularly in the case of skin- and mucosa-containing allografts, presents a commonly cited challenge for achieving allograft tolerance, as demonstrated by the improved allogenicity of VCA grafts following pre-transplantation depletion of donor leukocytes [48].

### 4.2. Allograft Rejection Rates in VCA

The incidence of acute rejection episodes in VCA have been outlined above, which far exceed that of solid organ counterparts (89% vs. 10–64%, respectively) [38,49,50]. Lung transplantation is largely considered one of the most challenging SOTs from an immunological perspective, with 29% of patients experiencing acute rejection within 1 year, which, although high, is significantly less prevalent than the 89% reported in VCA [38,51]. Contrary to the high AR rates in VCA, chronic rejection (CR) is reportedly lower (11%) in comparison to lung transplantation, where 50% and 67% of lung allografts are reported to succumb to chronic rejection within 5 and 10 years post-transplantation, respectively [38,52]. However, this comparison is somewhat premature given the relative infancy of VCA and the resultant paucity of long-term graft outcome data. The potent allogenic response, which too often complicates lung transplantation, is reportedly related to the high density of mucosal lymphatic tissue and its resident dense population of immune cells, in conjunction with constant exposure to external infective and inflammatory stimuli [53,54]. The lung presents many parallels to skin- and mucosa-containing allografts, with respect to their immunocyte density and interaction with the external environment. Many researchers posit that the characteristic skin and mucosal changes, which become externally visible during the early stages of rejection, likely result in a higher proportion of rejection episodes being diagnosed in VCA in comparison to SOT [24,55]. However, a dense population of immunocytes does not necessitate an effector response to allogenic tissue. The density of naïve T-lymphocytes in the lung, for example, presents a pool capable of transformation to Forkhead Box P3 positive (FOXP3+) regulatory T-cells (T_REGs_), if provided with the appropriate co-stimulation milieu. T_REGs_ are indispensable in achieving allograft tolerance and immune homeostasis. They are potent inhibitors of antigen presentation, the T_EFF_ and B-cell response, through a host of antigen specific and non-specific mechanisms [56]. An increased proportional population of FOXP3+ T_REGs_ within the transplant micro-environment is essential for the induction of transplant tolerance [57].

### 4.3. Pre-Operative and Peri-Operative Optimisation Strategies

Human leukocyte antigen (HLA) incompatibility remains a key predictor of graft survival in SOT [58,59]. However, the degree of HLA-match in VCA remains extremely varied in reported studies [60]. Although HLA mismatch is not a contraindication to transplantation, it remains an important precipitant to alloreactivity [61]. Pre-operative donor–recipient matching has improved graft survival in SOT, but is not of equal relevance to all organs [58]. The role of HLA mismatch, in liver transplantation, for example, is reportedly less impactful on allograft outcomes than in kidney, heart, or lung transplantation [58]. The infrastructure for pre-transplant HLA typing is presently available on an international scale, but the role of HLA in VCA requires further delineation. The management of extensive soft-tissue injuries, which may involve blood product transfusion or cadaveric grafting, may result in a higher proportion of VCA recipients being pre-sensitised and the subsequent development of anti-HLA antibodies [62].

### 4.4. Ischaemic Reperfusion Injury and Tissue Preservation

Tissue ischaemia results in cellular apoptosis and necrosis through the production of toxic metabolites and reactive oxygen species [63,64]. Reperfusion following prolonged ischaemia further worsens electrolyte dyscrasias, the production of toxic metabolites, and subsequent tissue damage, such is the basis of ischaemia reperfusion injury (IRI) [65]. IRI is an important factor of consideration in composite grafts, as the many discrete tissue types display varied tolerance for ischaemic conditions. For example, skin and subcutaneous tissue are quite resilient to hypoxia and tolerate cold ischaemic time up to three times longer than skeletal muscle [66,67,68]. Vascular endothelium is notoriously susceptible to thrombosis following the release of reactive oxygen species-mediated platelet aggregation and complement activation [69]. The advent of machine perfusion (MP) devices has been highly effective in solid organ transplantation, with liver allografts stored utilising MP demonstrating higher graft viability and significantly lower graft injury rates [70]. At present, concerted pre-clinical efforts are being made toward enhancing understanding of the effects of IRI on composite grafts and in evaluating the potential role for MP devices to ameliorate VCA outcomes [71,72,73].

### 4.5. Systemic Immunosuppression Dosage Required and Associated Morbidity

VCA differs from SOT with respect to its purpose, where VCA grafts are aimed at functional restoration and SOT represents a life-saving intervention for patients with end-stage organ disease. As such, the cost–benefit of systemic immunosuppression, given the potential for increased infective, neoplastic, and lymphoproliferative morbidity, requires more careful consideration in VCA [74,75]. The risk of non-melanomatous skin cancer alone in transplant recipients is at least 65 times that of their non-immunosuppressed counterparts, with the behaviour of these cutaneous malignancies being significantly more aggressive [76,77]. This is of particular relevance considering that the mean reported doses of immunosuppressive agents required in upper extremity and facial VCA are reportedly greater than in SOT [25]. However, in the case of SSF transplantation, the levels of immunosuppression required are reportedly equivalent to that of SOT alone [2]. The difference in immunosuppressive requirements in upper extremity/facial VCA and in SSF presents a somewhat contradictory finding. Some authors posit that the relative complexity of composite grafts, such as in upper-limb VCA, which contain osseous, tendinous, and neurological components, may amplify the immunogenicity of these grafts [78]. The lack of consensus regarding optimal immunosuppressive protocols in VCA and the relative infancy of the acute and chronic VCA rejection data further confounds this comparison.

## 5. Novel Immunomodulatory Approaches in Allotransplantation

### 5.1. Adoptive Cellular Therapies

Adoptive cellular therapies (ACTs) are being trialled with promising success in SOT [79]. Both innate and adaptive immune cells have demonstrated their potential immunomodulatory capacity as ACTs in SOT, with mesenchymal stromal cells, regulatory myeloid cells, and regulatory T-lymphocytes being among the favoured cell types [80,81,82,83,84,85]. The ONE and the TWO study have demonstrated clinically important effects of ex-vivo expanded T_REG_ therapies for immunomodulation in kidney transplantation [86,87]. Similarly, promising findings have been demonstrated in liver transplantation [85]. However, ex-vivo expansion of T_REGs_ is not without its limitations; namely, the relative paucity of T_REGs_ in peripheral blood, sample contamination by other CD4+ T-lymphocytes, and the paucity of Good Manufacturing Practice (GMP) expansion protocols [88,89,90,91]. The promise of ACTs to supersede modern immunosuppressive drugs and their associated morbidity represents a promising solution to many of the limitations of VCA. The degree to which the immunological mechanisms underpinning ACT-driven immunomodulation in SOT are understood facilitates progression at a rate greater than that currently achievable in VCA. Although the advancements in SOT present definite parallels, there are critical differences with regards to the immunogenicity of composite allografts, which demand further VCA specific trials. Although the immunogenicity of skin- and mucosa-containing VCAs pose a challenge, some VCA components may incur advantage from an immunomodulatory standpoint. Bone marrow (BM)-containing VCAs, for example, allow for the co-transplantation of passenger donor-specific haematopoietic progenitor cells, without the need for ex-vivo expansion. Vascularised BM transplantation has demonstrated the capacity to induce tolerance through mixed chimerism, prolonging graft survival in animal models [92,93,94]. The quality of evidence supporting the role of vascularised BM-induced tolerance in human VCA cohorts remains comparatively poor, limited to case series and observational studies, which lack both a comparator group and fail to report objective measures of chimerism [95].

### 5.2. Biomaterial-Based Immunoregulatory Strategies

In the case of VCA, the density of immunocytes, particularly within skin- and mucosa-containing allografts, may provide viable immunomodulatory targets, which supersede the need for ex-vivo expansion. However, the systemic administration of immunomodulatory agents, such as interleukin 2 (IL-2) or transforming growth factor beta (TGF-B), remain poorly tolerated, with significant off-target side-effects [96,97]. However, local administration has demonstrated clinical efficacy in a broad range of clinical applications, from wound healing to cartilage remodelling [98,99]. Biomaterials offer an advanced, modular toolbox capable of achieving highly controlled release of therapeutic agents whilst minimising off-target effects [97,100,101]. Achieving therapeutic effect can be challenging when delivering chemokines, growth factors, and/or interleukins, given the narrow therapeutic index of many of these agents and the severity of associated adverse reactions [102]. Biomaterials may be utilised to deliver controlled dosages of immunosuppressive drugs or immunoreactive cytokines/interleukins to the transplant micro-environment. The compositional and mechanical characteristics of these biomaterials are highly modifiable and can also significantly affect cellular behaviour [103].

Numerous pre-clinical animal studies have demonstrated capability in harnessing the power of biomaterials for the staged, localised delivery of bioactive modules capable of localised conversion of naïve CD4+ T-lymphocytes to FOXP3+ T_REGs_ with potent immunomodulatory capacities [104,105,106]. Fisher et al. utilised micro-nanoparticles as a delivery mechanism for rapamycin, IL-2, and TGF-Beta in a rat hindlimb transplant model. This biomaterial-based strategy demonstrated increased T_REG_ numbers and immunosuppressive activity within the allograft and draining lymphatic basin, resulting in allograft survival to the study endpoint at 300 days, despite the discontinuation of systemic immunosuppression [104].

More recently, micro-nanoparticles have been utilised to form artificial antigen presenting cells (aAPCs), which have demonstrated capacity to induce allorecognition and response mechanisms in-vivo [106]. The key applications of biomaterials for allotransplantation have been summarised (Table 1).

## 6. Latest Advancements, Ethical Considerations, and Future Directions

Progress in VCA is fundamentally reliant on the improvement of allograft survival. However, as with all emerging surgical entities, there remains significant debate with respect to the ethical implications of VCA for both the donor and recipient [27]. Overlooking the broader ethical context risks generating a non-feasible entity. This section outlines the latest advancements in VCA, the ethical considerations unique to VCA, and the likely future directions of the field.

### 6.1. Latest Advancements

The high throughput of scientific research, which has driven progress in SOT, provides a significant knowledge base from which VCA can advance. However, if VCA is to make meaningful progress, a concerted effort must be made to develop both preclinical and clinical research aimed at elucidating the immunological complexities of VCA. Recently, several groups have published impactful insights into VCA immunology utilising pre-clinical animal models. Kiefer et al. developed a rat hind-limb VCA model, which demonstrated the critical role of c-reactive protein (CRP) in VCA rejection through activation of non-classical monocytes [115]. The group further demonstrated the therapeutic potential of CRP stabilisation in attenuating acute allograft rejection [115]. Zhang et al. performed transcriptome profiling utilising a swine model to outline gene expression patterns during VCA rejection, generating numerous potential avenues for improved diagnostic and therapeutic strategies in VCA [116]. Goutard et al. demonstrated the superiority of sub-normothermic machine perfusion to static cold storage in VCA utilising a swine model, which was correlated using histopathological assessment of VCA biopsies [72]. Such pre-clinical research demonstrates promising progress in the field of VCA. Although ongoing research aims to delineate much of the immunological barriers to success in VCA, reconstructive surgeons continue to pioneer novel VCA subtypes capable of achieving functional and aesthetic restoration [117]. One such emergent VCA entity is the pioneering whole-eye transplant (WET), which aims to simultaneously restore sight and the deficient structural anatomical components, which are often lost in combination with severe facial injuries [118].

### 6.2. Ethical and Regulatory Considerations

The classification of VCA as an organ transplant has led to its regulation under the United States Department of Health and Human Services (DHHS) and the United Network for Organ Sharing (UNOS) [119]. Unlike solid organ transplants, VCA requires explicit consent from next-of-kin, even if the donor was a registered organ donor [120]. This raises ethical concerns, particularly in cases of facial or hand transplantation, where the identity-linked nature of the graft may affect donor families and recipients [121].

Internationally, VCA regulation varies. In the European Union, the 2010 Organ Directive provides a broad framework, but individual countries implement their own policies. France, a pioneer in VCA, mandates rigorous psychiatric evaluations and long-term follow-up for recipients. Spain has fully integrated VCA into its national transplant program, whereas Germany continues to develop its regulatory infrastructure. In contrast, countries such as India and Brazil have successfully performed VCA procedures, but lack standardised legal frameworks [122].

In the United Kingdom, VCA is governed by the Human Tissue Act 2004, which regulates the removal, storage, and use of human organs and tissues. The Human Tissue Authority (HTA) ensures compliance with ethical standards and proper consent procedures. The UK has performed several VCA procedures, including hand transplants, and continues to expand its expertise in the field.

Ireland is in the process of refining its legal framework for organ and tissue transplantation. The Human Tissue Bill aims to establish comprehensive regulations on the removal, retention, storage, and use of human tissue from deceased individuals, including provisions relevant to VCA [123]. However, specific legislation dedicated to VCA remains under development [122].

### 6.3. Future Directions

Despite its advancements, VCA faces several challenges that must be addressed to improve long-term outcomes. Key areas for future research include (Table 2):

## 7. Conclusions

VCA presents an exciting opportunity for the surgical provision of functional and anatomical restoration to patients whose reconstructive needs exceed the capabilities of autologous tissue transfer and prostheses. Although the past two decades have demonstrated significant progress in VCA, with growing global numbers of successful uterine, abdominal wall, facial, and upper-extremity transplantation, there remains significant opportunity for further advancement across immunological, biomedical engineering, and regulatory domains.

## Figures and Tables

**Table 1:** Summary of key biomaterial-driven immunomodulatory strategies in the allotransplantation literature. NP = Nanoparticle. MNP = Micro-nanoparticle. PLGA = Poly(lactic-co-glycolic acid). aAPC = Artificial Antigen Presenting Cell.

	Biomaterial	Therapeutic Agent	Application	Model	Result	Reference
**Drug-Eluting Biomaterials**	PLGA MP	Tacrolimus	Liver Transplant	Animal (Rat)	Stable release of tacrolimus and achieved steady state of immunosuppression for 10/7 following a single subcutaneous injection	Miyamoto 2004 [107]
	Acetylated Dextran MNP	Rapamycin	Transplant	In Vitro	Macrophages induced NO production equal in macrophages treated with Acetylated Dextran RAPA MPs and free RAPA	Kauffman 2012 [108]
	PLGA MP	Rapamycin	Transplant	In Vitro	RAPA MNPs show capacity for intracellular delivery to DCs, resulting in decreased immunostimulatory capacity	Forrest 2006 [109], Jhunjhunwala 2009 [110], Haddadi 2008 [111]
	Chitosan/PLA MP	Rapamycin	Corneal Transplant	Animal (Rabbit)	Median graft survival time greater in MP RAPA-treated rabbits versus free RAPA suspension	Yuan 2008 [112]
	PLGA MP	Rapamycin	Transplant	In Vitro	Stable release of RAPA-induced TREG dominant phenotype in vitro	Jhunjhunwala 2012 [113]
	Hydrogel	Tacrolimus	Osetomyocutaneous VCA	Animal (Swine)	Prolonged VCA survival with reduced intra-graft T-cell and neutrophil infiltration.	Hoyos 2024 [29]
**Cytokine-Eluting MNPs**	PLGA MP	IL2, TGFB (+RAPA)	Transplant	In Vitro	IL2/TGFB/RAPA MPs show synergistic effect in inducing naïve T Cell transformation to TREG, which outperformed soluble IL2/TGFB/RAPA	Jhunjhunwala 2012 [113]
	PLGA MP	IL2, TGFB (+RAPA) = “TRI-MP”	Hind Limb Transplant	Animal (Rat)	TRI-MP prolonged allograft survival to study end point and increased TREG number and function in graft and in draining lymph nodes	Fisher 2019 [104]
**Artificial APCs**	PLGA aAPCs	Antibody-MP Conjugate	In Vitro	In Vitro	45-Fold enhancement in T-Cell expansion when PLGA aAPC is incorporated	Steenblock 2008 [114]
	PLGA aAPCs	Antibody-MP Conjugate	Transplant	Animal (Rat)	PLGA aAPCs capable of converting naïve T-cells to TREGs with potent immunosuppressive activity in local tissue and draining lymph nodes	Rhodes 2020 [106]

**Table 2:** Summary box detailing the key areas for pre-clinical and clinical research, which are likely to generate the future directions of VCA.

Key Research Area	Summary
**Standardisation of outcome reporting**	International registries must track long-term functional, immunological, and psychological outcomes in VCA recipients. The standardisation of reporting outcomes is essential to aid inter-centre collaboration and will provide the volume of data required to aid clinical decision making and accurately inform pre-operative discussion.
**Improved specificity of pre-operative donor–recipient matching**	The role of HLA mismatch in VCA requires further delineation. The infrastructure for pre-operative HLA matching exists and may be employed with greater specificity in VCA.
**Ischaemic reperfusion injury**	Improved understanding of the mechanisms in which ischaemic reperfusion injury impacts VCA outcomes, in conjunction with developing sophisticated perfusion models capable of limiting IRI in VCA is likely to improve allograft outcomes.
**Advanced understanding of VCA immunology**	The immunological complexities of VCA demand concerted pre-clinical and clinical research efforts to further delineate cellular and genetic pathways relevant to VCA.
**Advanced immunomodulatory strategies**	The development of novel immunomodulatory approaches capable of reducing VCA rejection rates whilst mitigating the morbidity associated with systemic immunosuppressive regimens is the most essential barrier to advancement in VCA.

## Data Availability

No new data were created or analyzed in this study.
